# Full Genotyping of a Highly Polymorphic Human Gene Trait by Time-Resolved Fluorescence Resonance Energy Transfer

**DOI:** 10.1371/journal.pone.0107310

**Published:** 2014-09-12

**Authors:** Edoardo Totè, Marco Lamperti, Maria Bondani, Domenico Salerno, Valeria Cassina, Luca Nardo

**Affiliations:** 1 Department of Science and High Technology, University of Insubria, Como, Italy; 2 C. N. R. (Consiglio Nazionale delle Ricerche), Institute for Photonics and Nanotechnology, Como, Italy; 3 Department of Health Sciences, University of Milan Bicocca, Monza, Italy; CNR, Italy

## Abstract

The ability of detecting the subtle variations occurring, among different individuals, within specific DNA sequences encompassed in highly polymorphic genes discloses new applications in genomics and diagnostics. DQB1 is a gene of the HLA-II DQ locus of the Human Leukocyte Antigens (HLA) system. The polymorphisms of the trait of the DQB1 gene including codons 52–57 modulate the susceptibility to a number of severe pathologies. Moreover, the donor-receiver tissue compatibility in bone marrow transplantations is routinely assessed through crossed genotyping of DQB and DQA. For the above reasons, the development of rapid, reliable and cost-effective typing technologies of DQB1 in general, and more specifically of the codons 52–57, is a relevant although challenging task. Quantitative assessment of the fluorescence resonance energy transfer (FRET) efficiency between chromophores labelling the opposite ends of gene-specific oligonucleotide probes has proven to be a powerful tool to type DNA polymorphisms with single-nucleotide resolution. The FRET efficiency can be most conveniently quantified by applying a time-resolved fluorescence analysis methodology, *i.e.* time-correlated single-photon counting, which allows working on very diluted template specimens and in the presence of fluorescent contaminants. Here we present a full in-vitro characterization of the fluorescence responses of two probes when hybridized to oligonucleotide mixtures mimicking all the possible genotypes of the codons 52–57 trait of DQB1 (8 homozygous and 28 heterozygous). We show that each genotype can be effectively tagged by the combination of the fluorescence decay constants extrapolated from the data obtained with such probes.

## Introduction

The completion of the Human Genome Project has evidenced a large number of subtle variations (polymorphisms) among the genomes of different individuals of the same population [1]. Gene polymorphism is responsible for the expression of many variants of a protein among different subjects of the same species. To be classified into polymorphism, variations of gene sequences must appear in at least 1% of the population. In the latter case, the genetic variants are called alleles. The great majority of DNA polymorphisms consist in single-base variations, i.e. single-nucleotide polymorphisms (SNPs) [1]. Most SNPs occur in introns, thus they are silent, *i.e.* they have no effect on the individual phenotypes. However, SNPs, though rarely being directly pathogenic, are frequently correlated to the occurrence of multigenic diseases [2]. Moreover, certain SNPs result in mutations of genes that are pivotal in tumorigenesis. An example is the tumor suppressor gene p53 [3]. More recently, SNPs have been also exploited as markers in pharmacogenomics [4]. For all the above reasons, the possibility to perform wide range population screening of the relevant polymorphic genes would provide a powerful tool for disease prediction.

A particularly interesting highly polymorphic locus within the human genome is the major histocompatibility complex, located in the short arm of chromosome 6. It comprises many genes coding for the human leukocyte antigen (HLA) proteins that, originally discovered for their role in the rejection of transplanted organs, play a crucial role in the triggering of the immune response against pathogens. Indeed, they guide the development and the activation of T lymphocytes. There are two types of HLA molecules, namely class I and class II, that activate functionally distinct T cell populations, CD8+ T cells and CD4+ T cells, respectively [5]. The expression of certain HLA molecules has been associated with the susceptibility to several autoimmune diseases, including celiac disease [6]–[9], multiple sclerosis [10], [11], Hashimoto thyroiditis [12], Graves disease [12], narcolepsy [13], selective immunoglobuline A (IGA) as well as common variable immunodeficiencies (CVI) [14], sarcoidosis [15], primary biliary cirrhosis [16], autoimmune [17] and viral [18] hepatitis, insulin-dependent diabetes mellitus (IDDM) [12], [19], [20]. For all the above pathologies, genetic markers are found in the DQB1 gene, namely in the short though highly polymorphic trait encompassing codons 52–57. The latter trait is manifested in eight allelic variants: DQB1-0201, DQB1-0301, DQB1-0501, DQB1-0302, DQB1-0402, DQB1-0502, DQB1-0602, and DQB1-0503, which in turn correspond to 36 different genotypes (8 homozygous and 28 heterozygous). At least for some pathologies there seems to hold a link between the risk/protection factors and the presence in position 57 of a neutral residue. The latter is encoded in sequences DQB1-0201 and DQB1-0302 (alanine), DQB1-0501 (valine), DQB1-0502 (serine) instead of an aspartic acid residue (provided by all the remaining sequences). However, the susceptibility patterns are peculiar to the different pathologies and not easily explainable on the mere basis of the coded aminoacids. A compendium of disease-correlated DQB1 genotypes and of their Asp/non-Asp nature is compiled in Table 1.

**Table 1:** Compendium of disease susceptibility/protection patterns correlated with DQB1 genotype.

Disease	Predisposing genotypes	Protective genotypes	Notes
Celiac disease	0201/0XXX; 0302/0302;0302/0501; 0302/0301	–	90% patients bear the 0201/0201 genotype
IDDM	0201/0XXX; 0302/0XXX	0602/0XXX; 0301/0XXX	–
IGA-CVID	Non-Asp 57 in both alleles	Asp 57 in both alleles	0201/0201 genotype is maximally predisposing
Graves disease	0201/0XXX	–	0201/0302 genotype is maximally predisposing
Hashimoto thyroiditis	0201/0XXX	0302/0XXX	–
Primary biliary cirrhosis	0402/0402	–	–
Sarcoidosis	0602/0XXX	0201/0XXX	–
Narcolepsy	0602/0XXX	–	0602/0602 genotype is maximally predisposing
Type 1 autoimmune hepatitis	–	0301/0XXX	
Viral hepatitis C	–	0502/0XXX	

From the above picture, it is clear that being able to determine the genotype of an individual with respect to the highly polymorphic DBQ1 gene trait coding for residues 52–57 would be desirable for various clinical purposes. The ambition of introducing wide-scale screening protocols calls for the development of rapid high-throughput DQB1 typing technologies. Nowadays, molecular typing of polymorphic genes may be performed with single nucleotide sensitivity by applying several different techniques [21]. However, as a rather common feature, virtually all these techniques require amplification of the gene trait to be typed by means of Polymerase Chain Reaction (PCR) as the initial step. Although it is out of doubt that PCR has revolutionized biology, it is not the ideal technique to be applied to the screening of vast populations, due to lack of both readiness and cost-effectiveness. During the last decade, molecular typing techniques based on exploitation of the differential fluorescence emission of fluorophore-labeled allele-specific oligonucleotide probes upon recognition of their genomic targets have raised increasing interest because of their particularly neat response (i.e. high signal-to-noise ratio and low probability of false-positives detection), conferring to fluorescence-based assays notable readiness and ease of interpretation [22]–[27]. The common rationale of the majority of such techniques is that light emission by the fluorophore functionalizing the oligonucleotide probe (hereafter called the fluorescence donor, D) can be quenched by suitable dyes (hereafter referred to as fluorescence acceptors, A) in a strongly donor-acceptor-distance dependent way by virtue of a non-radiative decay mechanism of the D from the fluorescent excited state, called fluorescence resonance energy transfer (FRET). The latter occurs whenever the absorption spectrum of the A is significantly superimposed to the fluorescence emission spectrum of the D.

Among others [23], [24], FRET-based molecular typing methods making use of D-A dual-labeled probes have proven to be particularly sensitive [22], [25]–[27]. Such probes can be designed to be very slightly fluorescent while not annealed to their genomic target. In general, single-stranded DNA has a much shorter persistence length than double-stranded DNA. Thus, due to hydrophobicity of bases the non-hybridized probe coils in a compact structure in which the 5′ and 3′ ends (and thus the D and the A) are near. Upon hybridization to the target, the probe assumes a rigid longitudinal structure, and the D, brought further apart from the A, emits fluorescence. Palindromic sequences may be included at the opposite ends of a probe (in this case called Molecular Beacon) in order to stabilize the single-strand in a hairpin structure, in which fluorescence is optimally quenched. In most instances a number of allele specific probes equal to that of possible allelic variants are used. Annealing conditions allowing hybridization to the template DNA only of the exactly matching probe are then determined, and the response of a typing experiment consists in emission of a detectable fluorescence signal exclusively by the specimen added with such probe [22]. However, during the last five years, we validated an alternative approach based on quantitative analysis of FRET-tuned fluorescence signals [25]–[29]. In the latter approach, a reduced number of probes (ideally a single probe) are used, all of which being capable of hybridizing to any of the possible target sequences (i.e. to each possible polymorphic variant of the gene of interest) with sufficient selectivity with respect to other genomic regions. Allele-specific annealing thus occurs through formation of duplexes whose structural details depend on the probe-target degree of complementarity, thus, in the end, from the target sequence. Routinely, FRET efficiencies are evaluated through relative intensity measurements. Attempts of pursuing molecular typing of polymorphic genes by exploiting this method have been made [23], [24] and have brought to a proof of principle of the feasibility of detecting the polymorphisms of the ABL portion of the BCR-ABL oncogene [24]. However, this approach is plagued by major limitations. Firstly, quantitative assessments of the fluorescence intensity are biased by variations, among specimens, in the probe as well as in template DNA concentrations. Moreover, application to non-purified genomic material is complicated by superposition to the relevant fluorescence signal emitted by the probe of an unpredictable amount of fluorescence produced by endogenous fluorophores. Finally, detectors endowed with light-intensity-proportional responses must be used, whose low quantum efficiency makes PCR amplification hardly avoidable. The above-mentioned drawbacks can be overcome by quantitating the variations in FRET efficiency through measurements of the fluorescence decay time *τ_D_* of D. Indeed, *τ_D_* can be determined by means of time-correlated single-photon counting (TCSPC). TCSPC works by detecting and timing one photon per excitation pulse at most. The subsequent reconstruction of the statistical distribution of the detection times relative to the excitation pulses yields the fluorescence decay pattern, whose fitting provides the value of *τ_D_*. For this reason, TCSPC is intrinsically independent from the D concentration and takes advantage of the usage of Geiger-mode light detectors, such as single-photon avalanche diodes, which are sensitive to even extremely deem fluorescence pulses, virtually reduced to few/single photons.

We recently showed that by this method it is possible to pursue the recognition of the DQB1-0201 genotype, conferring susceptibility to, e.g., celiac disease and IDDM, by working on template DNA contained in untreated (i.e. unamplified and non-purified) cell extracts [27]. To this aim we designed an oligonucleotide probe dual labelled at its 5′ and 3′ ends, respectively, with a fluorescent dye and with a non-emitting FRET acceptor, and with sequence complementary to the region of DQB1-0201 allele encompassing codons 51–57 and the first nucleotide of codon 58. Namely, the fluorophore was tetramethyl-rhodamine (TAMRA) while the acceptor was black-hole quencher 2 (BHQ2). The latter probe, hereafter Probe1, was made to hybridized to oligonucleotides mimicking any of the eight possible allelic variants of that region of DQB1. The *τ_D_* of TAMRA attached to Probe1 annealed to each of the target oligonucleotides was measured with 30 ps resolution. Because each target oligonucleotide was characterized by distinctive mismatches with respect to Probe1, we expected the resulting probe/target duplexes to have slightly different conformations, hopefully reflected in significantly different *τ_D_* values. Indeed, the allele of interest, DQB1-0201, yielded a *τ_D_* value distinct from the most similar of the others for as much as 42 standard deviations. Moreover, we managed to reveal the presence of the DQB1-0201 allelic variant also in heterozygous conditions, and to discriminate the latter from the homozygous case. Furthermore, Probe1 demonstrated to be sufficiently selective to specifically recognize the DQB1 trait of interest within the whole genome. Finally, we were able to replicate the *τ_D_* results by using unpurified template DNA contained in bare cell lysates. However, we failed to obtain a full discrimination of alleles. Namely, even in the preliminary calibration measurements undergone on the oligonucleotide targets, Probe1 yielded hardly distinguishable *τ_D_* values when annealed to DQB1-0503, DQB1-0602, and DQB1-0402-like sequences. With the goal of breaking such a degeneracy on *τ_D_* values and pursuing a complete genotyping of the DQB1 trait of interest, more recently we performed additional TCSPC experiments aimed at characterizing the fluorescence decay of TAMRA attached to DNA duplexes obtained by annealing the same target oligonucleotides of above with a second oligonucleotide probe (Probe2), complementary to DQB1-0503 [28]. We obtained notably different *τ_D_* values for Probe2 hybrids with DQB1-05031, DQB1-0602, and DQB1-0402-like targets.

In this work we present a systematic study of the fluorescence decay patters produced by Probe1 and Probe2 upon reaction with oligonucleotide samples mimicking all the possible genotypes of the DQB1 trait encompassing codons 52–57 (i.e. 8 samples containing a single target oligonucleotide variant and the 28 samples resulting by mixing 1∶1 concentrations of any possible pair of oligonucleotides). We demonstrate that, while the “homozygous” samples can be discriminated by cross-combining the *τ_D_* value obtained with Probe1 with that obtained with Probe2, for the “heterozygous” ones it is still possible to extract an average decay time value from the decay data obtained with both probes, <*τ_D_*>. The (<*τ_D,Probe1_*>; <*τ_D,Probe2_*>) pair are an exhaustive tag-parameter allowing to discriminate among the different heterozygous genotypes.

## Materials and Methods

### Oligonucleotides and Sample Preparation

**Figure 1:**
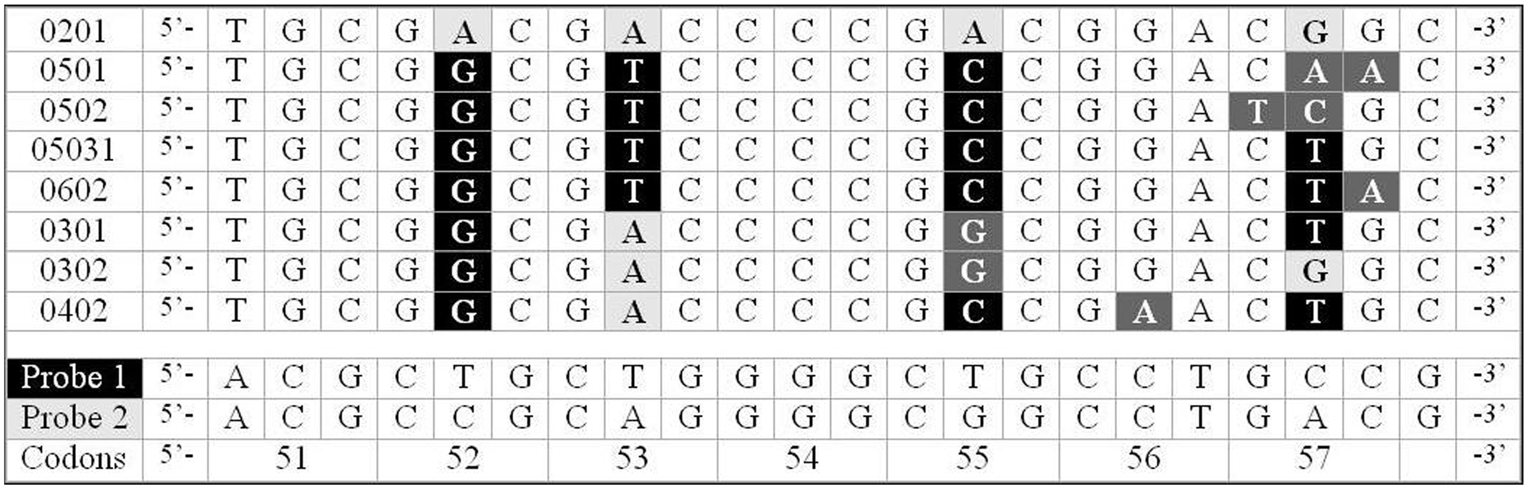
Sequences of the eight target oligonucleotides and of the TAMRA-BHQ2 dual-labelled probes 1 and 2. Mismatch-sites of the target oligonucleotides with respect to Probe1 are black-shaded, those with respect to Probe2 are light-gray shaded. Mismatching sites common to both probes are indicated by dark-grey shading.

In Figure 1 the eight target oligonucleotides reproducing the possible sequences of the 22-bases polymorphic trait encompassing codons 51 to 57 and the first base of codon 58 are depicted. In the same figure, the TAMRA-BHQ2 dual-labelled probes Probe1 and Probe2, with sequences complementary to the DQB1-0201 and DQB1-0503 allele-mimicking oligos, respectively, are also shown. Mismatch-sites of the target oligonucleotides with respect to Probe1 are black-shaded, while those with respect to Probe2 are light-gray shaded. Mismatching sites common to both probes are indicated by dark-grey shading. The target oligonucleotides were purchased by TIB Molbiol (Genova, Italy) and were desalted and further purified by double reverse-phase procedure. The dual-labelled probes were purchased by Eurofins MWG Operon (Ebersberg, Germany) and purified by high-performance liquid chromatography. A nominal amount of 20 nanomoles of the lyophilized oligonucleotides was resuspended in appropriate volumes (indicated by the suppliers) of Tris-HCl EDTA buffer at pH 7.6 and 10 mM ionic strength, to obtain 50 µM concentrated solutions. Equal amounts (10 µL) of probe and target stock solutions were mixed to obtain the “homozygous” solutions. To attain the solutions mimicking the heterozygous genotypes, e.g. DQB1-0XXX/DQB1-0YYY, 5 µL of DQB1-0XXX target oligo and 5 µL of DQB1-0YYY were mixed to 10 µL of probe. The annealing protocol, which is fully described elsewhere [27], consisted in heating the samples to 98°C in order to denaturate any residual secondary structures of the single-stranded oligonucleotides, cooling at the rate of 1°C/min to the annealing temperature and allowing the probe to react with the complementary oligonucleotide for 10 minutes. For “heterozygous” samples, the cooling was stopped for 10 minutes at the annealing temperatures of both targets. Finally, the solutions were cooled to 25°C at 3°C/min rate. The thermostat of a ThermoQuest-Finnigan gas chromatograph (San Jose, CA, USA) was used as the temperature controller. The obtained solutions were diluted in phosphate buffer at pH 7.6 and 150 mM ionic strength to a final probe concentration of 250 nM, to produce the sample for TCSPC analysis.

### Time-Correlated Single-Photon Counting Apparatus

The fluorescence excitation/detection apparatus is extensively described elsewhere [26]. Briefly, a 113 MHz repetition rated train of excitation pulses (pulse duration 6.4 ps) at 532 nm is provided by a Nd: VAN cw-mode-locked laser (GE-100, Time Bandwidth Products, Zurich, CH) and attenuated by a factor of 10^3^ by means of neutral density filters, in order to achieve single-photon regime (photon counts rate <100 kHz for all samples). Fluorescence is collected at 90° to the excitation beam through a long-wavelength-pass filter with cut at 550 nm, and focused onto the sensitive area of a single-photon avalanche diode (PDM50, Micro-photon-devices, Bolzano, IT) by a 20X microscope objective. The avalanche pulses give the START signal to an integrated TCSPC board (which is a single card of a SPC 152 module, Becker & Hickl, Berlin, DE). The STOP signal is provided by the subsequent laser pulse, as detected by a fast photodiode internal to the laser. The START-STOP lag-times occurring in a time window of 9 ns are digitized with 2.44 ps/channel resolution. The full-width at half-maximum duration of the detected excitation pulse is <30 ps.

### Data Analysis

**Figure 2:**
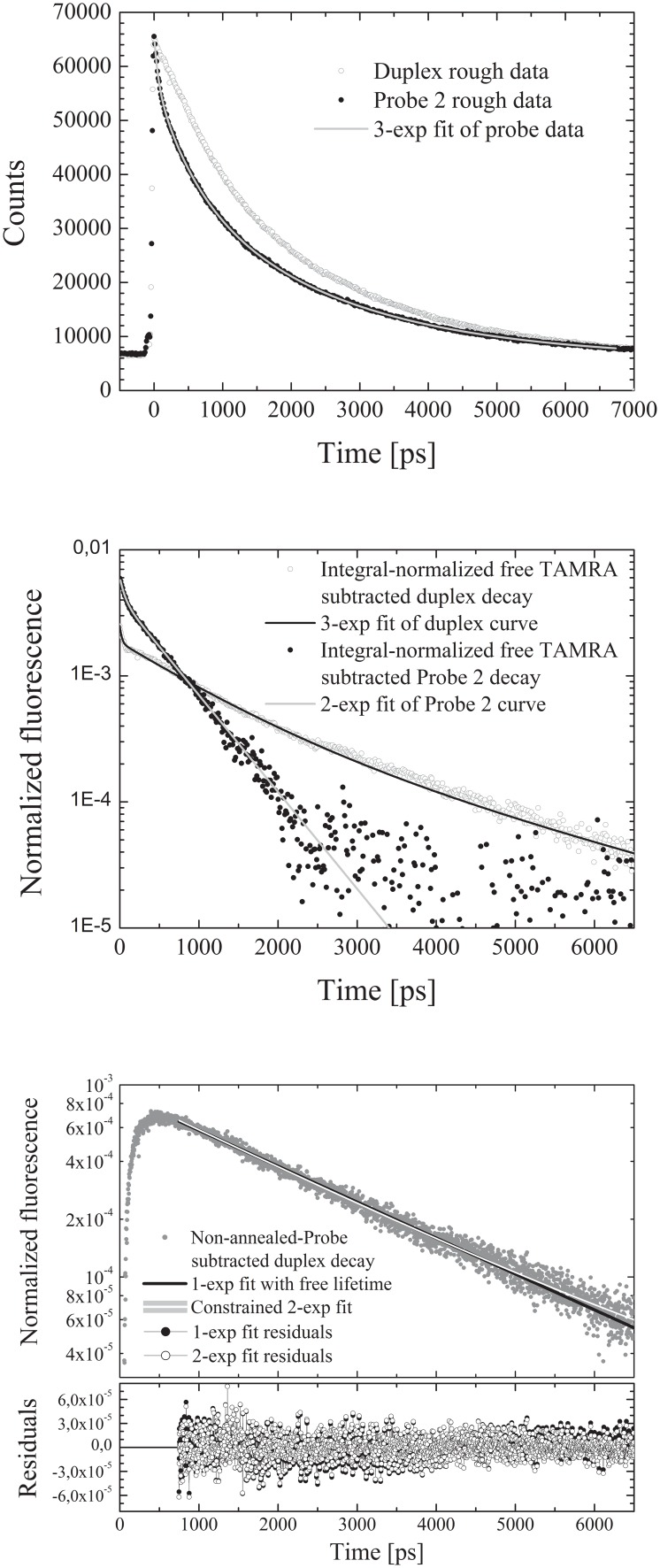
Main steps of the data cleaning procedure shown for an exemplary dataset, namely one of those obtained with the DQB1-0201/0301 genotype mimicking oligonucleotide mixture annealed to Probe2. Upper panel: the decay patterns of the single-stranded probe and of the duplex are synchronized and the probe decay is fitted to a three-exponential-components model. Central panel: the longest lived component of the fit, which is attributed to non-quenched TAMRA emission, is subtracted to both the probe and the duplex distributions, which are then normalized to their integrals and fitted to a 2-exponential and a 3-exponential model, respectively. Lower panel: the weighted subtraction of the non-hybridized probe fraction to the duplex decay yields the net decay of TAMRA molecules attached to intact (i.e. bearing also BHQ2) and correctly hybridized probes. The latter can either be interpolated with a single-exponential or fitted to a constrained 2-exponential model in which the decay time constants values are fixed to those measured for any pair of alleles, the relative amplitudes are allowed to vary and their values analyzed in order to derive the correct genotype (see text for details). In the figure the 2-exponential fitting curve obtained by using the decay time constant values pertaining to the correct pair of alleles is reported.

The decay patterns of all the probe/target duplex samples and of the single-stranded probe were measured for 5 minutes. The data processing protocol devised in order to get read of the contributions to the fluorescence decays of unquenched TAMRA molecules and non-hybridized (*i.e.* single-stranded) probe molecules, which is detailed in previous works [25]–[27] and briefly summarized in Fig. 2, was standardized and automatized by compiling a Matlab routine. We further subtracted a constant background value accounting for non-time-correlated environmental light, which was evaluated by acquiring a photon lag-times pattern with the sample substituted by a water-filled cell for the same measuring time, and averaging the counts values over 1000 channels far from the pulse response peak. Preliminary to the new experiments on heterozygous genotypes mimicking samples, we repeated (in one parallel only) measurements on homozygous genotypes mimicking samples. The so obtained data, after being “cleaned” by using the Matlab routine, were fitted to single exponential decays such as in previous works [27], [28].

The “clean” heterozygous data ideally represent the decay distributions built up uniquely by the fluorescence photons emitted by TAMRA molecules labelling intact (i.e. BHQ2-functionalized) probes correctly hybridized to a target oligonucleotide. Two different approaches were applied to analyze such decays. Firstly, two-exponential fits were performed, with time constants fixed at any possible pair of *τ_D_* values measured for homozygous alleles. Ex-post discrimination of the correct genotype by comparison of the results of the above fits was attempted. Secondly, the “clean” heterozygous decay distributions were fitted to single exponential decays. The extracted *τ_D_* values, representing a sort of average decay constants, were used to tag each genotype. In both cases, due to the negligible spread of the instrumental response function (IRF, <30 ps) with respect to the measured *τ_D_* values (>2 ns), no IRF deconvolution algorithm was applied. However, the fitting interval was chosen to be of 2800 channels, starting from 200 channels (i.e., 488 ps, thus 16 IRF full-width at half maximum) after the detected excitation pulse peak. This choice, besides assuring to start the fitting far enough from excitation in order to be able to bypass deformations induced in the decay histograms by convolution with the IRF, allowed to exclude the board non-linearity region (i.e., the transient in which the current flowing through the board time-to-amplitude converter switches on and off).

It should be mentioned that putative systematic errors might arise from an incorrect choice of the fitting interval. However, fitting parameters values stable within few percent were obtained for exemplary datasets upon variation of the initial channel of the fitting interval in the range from 170 to 600 channels after the detected excitation pulse peak, with the last channel fixed at 3000 channels after excitation. Moreover, because our measurements are differential in nature and the *τ_D_* values are simply used as tags of the corresponding genotypes throughout the manuscript, without attempting any absolute evaluation of the donor-to-acceptor distance, systematic errors of any source would not invalidate our conclusions.

All the experiments on heterozygous samples were performed in triplicate, and the values reported below are the average on the three parallel samples. The pertaining standard deviations were used as experimental errors. The built-in Matlab fitting tool which we exploited within our routine automatically yields confidence intervals for the fitting parameters values, which were in all cases smaller than such standard deviations.

## Results and Discussion

Firstly, we shortly report on the results yielded by analysis of the repeated experiments on homozygous samples. The *τ_D_* values obtained from the new datasets were equal, within the experimental errors, to those published in the past. This stems in support of both the repeatability and reliability of the typing protocol and the correctness of the new automatized data processing routine. The *τ_D_* values resulting by averaging the old with the new fitting parameters, with errors given by standard deviations, are summarized in Table 2. The new data are reported in parenthesis, while the old ones are published in [28].

**Table 2:** Fluorescence decay time, *τ_D_*, of the TAMRA fluorophore linked to Probe1 and Probe2 hybridized to oligonucleotides mimicking all the allelic variants of the DQB1 codon 51–57 trait (cumulative average of previously published data [28] with those derived in the check experiments presented here, which are reported in parenthesis.

DQB1-Allele-mimicking oligo	*τ_D_* (Probe1)*±σ*(*τ_D_*) [ps]	*τ_D_* (Probe2)*±σ*(*τ_D_*) [ps]
DQB1-0201	2725*±*3 (2724)	2562*±*7 (2560)
DQB1-0501	2514*±*3 (2512)	2200*±*12 (2196)
DQB1-0502	2404*±*10 (2406)	2031*±*3 (2033)
DQB1-05031	2427*±*3 (2430)	2398*±*3 (2402)
DQB1-0602	2431*±*8 (2434)	2194*±*5 (2196)
DQB1-0301	2480*±*3 (2478)	2137*±*4 (2139)
DQB1-0302	2599*±*3 (2600)	2489*±*3 (2487)
DQB1-0402	2462*±*3 (2464)	1970*±*2 (1972)

We now come to describe in details the results obtained on the heterozygous genotype mimicking samples. Theoretically, for both probes and for any given “heterozygous” sample DQB1-0XXX/DQB1-0YYY, after subtraction of the unquenched TAMRA and non-hybridized probe transients and of the constant background, there remain only two exponential components building up the “cleaned” fluorescence decay. The first component is produced by the part of the probe annealed to the target oligonucleotide bearing the sequence of the DQB1-0XXX allele. This should decay with the time constant *τ_D,_*_XXX_ tagging the homozygous DQB1-0XXX genotype, reported in Table 2. The other component is due to the emission of the remaining probe molecules, which are hybridized to oligonucleotides reproducing DQB1-0YYY and thus decay with the time constant *τ_D,_*_YYY_ tagging DQB1-0YYY in the same Table. In practice, fitting the “heterozygous” datasets to a double exponential without introducing constrains leaded to non-convergence. More specifically, the two time constants tended to approach a common value <*τ_D_*>, being min(*τ_D,_*_XXX_; *τ_D,_*_YYY_) < <*τ_D_*> <max(*τ_D,_*_XXX_; *τ_D,_*_YYY_), which can be interpreted as a sort of average decay time, while the equilibrium values of the relative amplitudes were not reached. We attribute this fact to the rather similar (although statistically differentiable) time constants tagging the different allelic variants. Nonetheless, for all the “heterozygous” samples, fitting the “cleaned” decay distributions to a single exponential model yields fits significantly less accurate than those obtained for the homozygous-genotypes-mimicking samples (see [29] for a detailed discussion on this topic). Furthermore such single-exponential fits yield <*τ_D_*> values very similar to those obtained by means of the unconstrained double-exponential fit and not ascribable to any of those reported in Table 2. This result allows us to conclude that our decay distributions are sensitive to the homozygous/heterozygous nature of the template DNA to be typed.

We thus implemented a data analysis procedure based on constrained double-exponential fit of the data pertaining to the heterozygous samples. Namely, for a subset of 10 out of these datasets we performed 28 fits, in which the time constants were fixed successively to any possible pair *τ_D,_*_XXX_; *τ_D,_*_YYY_ of *τ_D_* values measured for homozygous alleles, and the χ^2^-value was minimized leaving only the values of the corresponding initial amplitudes *f*_XXX_ and *f*_YYY_ as free fitting parameters. A first non-trivial result of this procedure, which is worth being mentioned, is that, upon fixing the *τ_D,_*_XXX_ and *τ_D,_*_YYY_ values to those proper of the correct allelic pair, fits significantly more accurate than single-exponential fits with free <*τ_D_*> are obtained (see Fig. 2c), particularly the bad agreement between the single-exponential fitting curve and the tail of the experimental decay pattern). Moreover, as discussed at length in the File S1, in which we also provide all the fitting results obtained for the exemplary genotype DQB1 0201/0502, most of the heterozygous genotypes can be excluded on the basis of compelling physical arguments on the retrieved initial amplitude values. However, for all the ten probed genotypes, the fitting with the *τ_D,_*_XXX_ and *τ_D,_*_YYY_ values proper of the correct genotype yields physically meaningful amplitudes values for both probes. In Table 3 we report the *f*_XXX,Probe1_; *f*_YYY,Probe1_; and χ^2^ values obtained for the genotypes surviving to the constrained fit analysis with both Probe1 and Probe2 for the exemplary DQB1 0201/0502 dataset. The corresponding values for Probe2 are reported in Table 4. Notably, for both probes the *τ_D,_*_XXX_; *τ_D,_*_YYY_ pair yielding the best χ^2^ value is *τ_D,_*_201_; *τ_D,_*_502_, i.e. the fit is maximally accurate when the correct genotype is assumed. However, in this exemplary case (and in seven others out of the ten scrutinized), a few other genotypes (three in the case under examination of the DQB1-0201/DQB1-0502 sample) survive the self-consistency analysis on the relative amplitudes values and yield fits of comparable (although somewhat worse) accuracy. In other terms, a univocal attribution of genotype is not feasible by means of this analysis, which was thus abandoned.

**Table 3:** Initial amplitudes and χ^2^ values yielded by the fitting of the Probe1 data for the genotypes surviving the constrained fit analysis with both probes.

Genotype	f_xxx_×10^4^	f_YYY_×10^4^	χ^2^	<τ_D_>	F_xxx_
0201/0502	3.1425	2.8774	0.9791	2571.57	0.52
0302/0602	5.0585	0.9528	0.9806	2572.21	0,84
0302/0301	4.6532	1.3574	0.9807	2572.35	0.77
0302/0501	4.1317	1.8785	0.9808	2572.43	0,69

In the last two columns the calculated values of the average lifetime and fraction of probe hybridized to the DQB1-0XXX allele are reported.

**Table 4:** Initial amplitudes and χ^2^ values yielded by the fitting of the Probe2 data for the genotypes surviving the constrained fit analysis with both probes.

Genotype	f_xxx_×10^4^	f_YYY_×10^4^	χ^2^	<τ_D_>	F_xxx_
0502/0201	5.3401	4.6075	0.9789	2276.87	0.54
0301/0302	5.8767	4.0303	1.0057	2280.19	0.59
0602/0302	6.9683	2.9263	1.0094	2281.25	0.70
0501/0302	7.1325	2.7607	1.0099	2281.37	0,72

In the last two columns the calculated values of the average lifetime and fraction of probe hybridized to the DQB1-0XXX allele are reported.

Interestingly, the average decay time values <*τ_D_*> (see fifth column in Tables 3 and 4) calculated from any of the surviving set of fitting parameters as 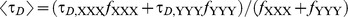 are very similar, and equal within the experimental errors to the <*τ_D_*> values yielded by both single exponential fit (2566±9 ps and 2279±11 ps for the DQB1-0201/DQB1-0502 sample and Probe1 and Probe2, respectively) and unconstrained double exponential fits. This fact suggests that fitting convergence is mainly dictated by retrieval of the correct average decay time value. Hence, we decided to use the <*τ_D_*> value restituted by single exponential fit as the representative tag-parameter also for the heterozygous sample, in spite of the somewhat lower quality of fits, which in turn leads to higher uncertainties on the average <*τ_D_*> values calculated over the three parallel samples. The results of such single-exponential fits are reported in Table 5.

**Table 5:** <*τ_D_*> values resulting from single-exponential fit of the decay data obtained for Probe1 (second column) and Probe2 (fourth column) made to react with the “heterozygous” oligonucleotide mixtures.

Genotype	<τ_D,Probe1_>	σ	<τ_D,Probe2_>	Σ
0201/0501	2608	7	2376	12
0201/0302	2662	7	2553	11
0201/0502	2566	9	2279	11
0201/0503	2562	10	2474	8
0201/0602	2570	10	2379	9
0201/0301	2594	5	2344	5
0201/0402	2593	11	2247	10
0302/0402	2527	9	2223	6
0302/0501	2552	8	2335	7
0302/0301	2536	9	2308	4
0302/0502	2499	11	2264	7
0302/0503	2508	10	2440	4
0302/0602	2511	11	2334	5
0501/0502	2455	8	2111	7
0501/0503	2470	9	2288	10
0501/0602	2464	12	2196	9
0501/0301	2494	9	2155	10
0501/0402	2471	6	2075	10
0502/0602	2413	11	2099	6
0502/0503	2401	9	2203	6
0502/0301	2445	11	2076	6
0502/0402	2423	12	1995	4
0503/0301	2453	6	2252	5
0503/0602	2425	3	2280	8
0503/0402	2445	6	2169	6
0602/0402	2431	11	2062	8
0602/0301	2447	9	2128	6
0301/0402	2470	3	2044	7

**Figure 3:**
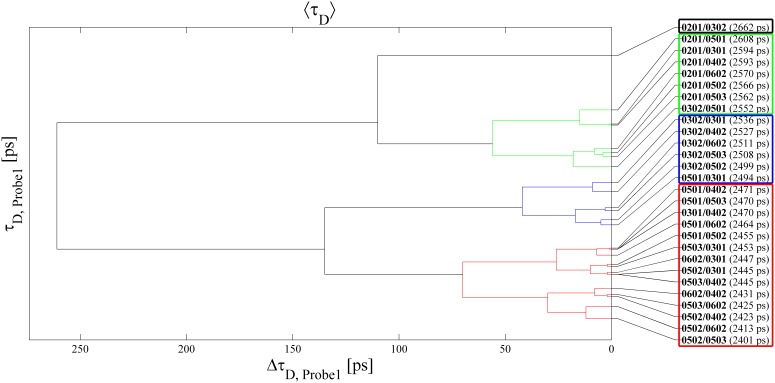
Dendrogram obtained by performing cluster analysis on Probe1 <*τ_D_*> values, applying Euclidean metrics and the complete-linkage cluster-joining criterion.

**Figure 4:**
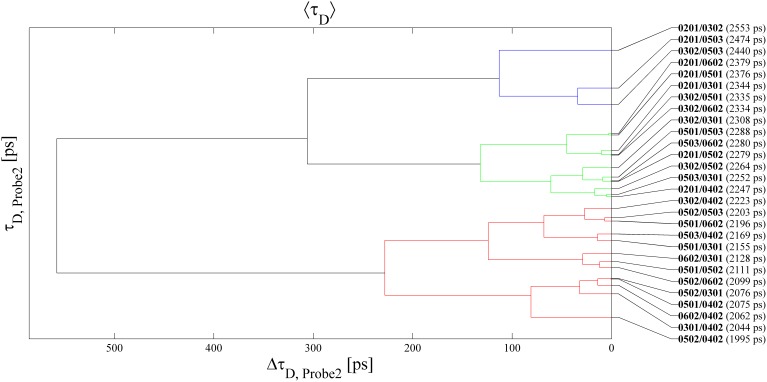
Corresponding dendrogram obtained with Probe2 <*τ_D_*> values.

We first comment on the results obtained with Probe1. Although the <*τ_D_*> values obviously do not allow unambiguous discrimination of the various heterozygous genotypes (the latter was not possible with this probe even limiting the analysis to the homozygous genotypes), relevant information concerning the Asp/non-Asp nature of residue 57 can be extracted from these data. In Fig. 3 we show the dendrogram resulting from cluster analysis of the Probe1 data obtained assuming Euclidean metric.

(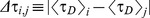) as the distance and applying the complete-linkage cluster joining criterion. In the latter, the distance between two clusters is defined as the maximum distance between one element belonging to the first and one element belonging to the second cluster. The heterozygous genotypes bearing both alleles coding for Asp residues cluster up in a common family (red branches of the dendrogram). Even more relevantly, the genotype associated to maximum proneness to Graves disease and to incremented proneness to celiac disease, *i.e.* DQB1-0201/DQB1-0302, clusters on its own (black branch). In addition, all and only the heterozygous genotypes conveying *per-se* susceptibility to celiac disease (i.e. DQB1-0201/DQB1-XXX and DQB1-0501/DQB1-0302) make up the second (green) family of the dendrogram. It is also worth noting that the genotypes containing the maximally disease-correlated allele DQB1-0201 (conferring susceptibility to celiac and Graves diseases, IDDM, Hashimoto thyroiditis, IGA, and CVID, and protection with respect to sarcoidosis) all cluster in the above two families. Finally, the blue cluster contains all the DQB1-0302/DQB1-0XXX genotypes different from DQB1-0201/DQB1-0302, and only one additional “extraneous” genotype, namely DQB1-0501/DQB1-0301. We recall that the presence of the DQB1-0302 allele is correlated to increased risk factor for IDDM, IGA and CVID and to protection with respect to Hashimoto thyroiditis. We also attempted cluster analysis of the Probe2 <*τ_D_*> values, but the results (reported in the dendrogram of Fig. 4) are less suggestive from a diagnostic standpoint, as the segregated families do not contain genotypes endowing with susceptibility to a common disease. Moreover, also with this probe unambiguous recognition of any possible genotype is not feasible (e.g. the DQB1-0501/DQB1-0302 genotype is not distinguishable from DQB1-0602/DQB1-0302, DQB1-0201/DQB1-0501 from DQB1-0201/DQB1-0602, DQB1-0201/DQB1-0402 from DQB1-0301/DQB1-0503, and DQB1-0501/DQB1-0402 from DQB1-0301/DQB1-0502, as evidenced by the negligible distance values visualized in the dendrogram).

**Figure 5:**
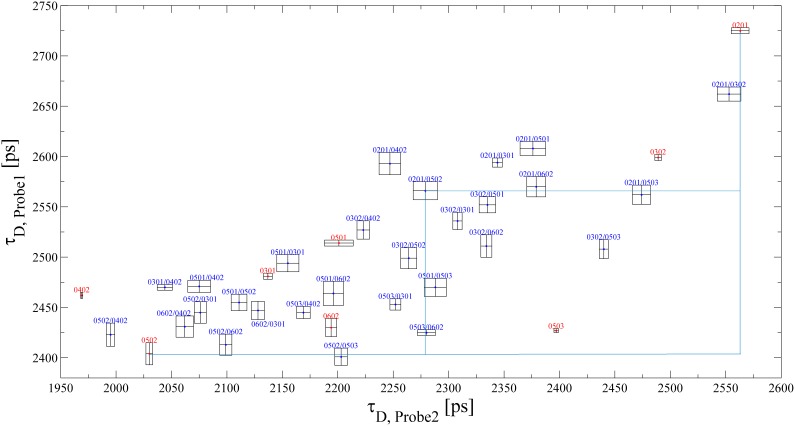
Cross combination of the <*τ_D_*> values obtained with the two probes allows discrimination of all possible DQB1 genotypes. Here, each genotype is represented by a point whose abscissa is given by the <*τ_D,Probe2_*> value and whose ordinate is <*τ_D,Probe1_*>. The half-base and half-height of the rectangles surrounding each point represent σ(<*τ_D,Probe2_*>) and σ(<*τ_D,Probe1_*>), respectively. Discrimination is warranted by lack of superposition among different rectangles.

The next step consisted in simultaneously exploiting the <*τ_D_*> data pertaining to both probes. With this aim, we devised the graph shown in Fig. 5, in which each genotype is represented by a point whose abscissa is given by the <*τ_D,Probe2_*> value and whose ordinate is <*τ_D,Probe1_*>. Moreover, for any heterozygous genotype DQB1-0XXX/DQB1-0YYY, the fraction F_XXX,Probe1_ of Probe1 hybridized to the DQB1-0XXX allele can be estimated graphically from Fig. 4. The relevant segments are indicated in Fig. 4 for the same DQB1-0201/DQB1-0502 sample also used to exemplify the constrained double-exponential fit procedure. Namely, F_XXX,Probe1_ (F_201,Probe1_ in the example) is found by dividing the difference (thick vertical segment in Fig. 4) of the ordinates Y_XXX,YYY_ and Y_YYY,YYY_ of the points corresponding to the heterozygous genotype (Y_201,502_ in the case under discussion) and to the homozygous DQB1-0YYY/DQB1-0YYY genotype (Y_502,502_ in the example) by the difference (thin vertical segment) between the ordinate Y_XXX,XXX_ of the point representing the homozygous genotypes DQB1-0XXX/DQB1-0XXX (Y_201,201_) and Y_YYY,YYY_. The same relation holds between the fraction F_XXX,Probe2_ of Probe2 hybridized to DQB1-0XXX and the abscissa of the above-mentioned points of Fig. 4. Notably, for all the 10 heterozygous samples which were submitted to constrained double exponential fit analysis, the so derived values of F_XXX,Probe1_ and F_XXX,Probe2_ (in the example F_201,Probe1_ = 0.50, F_502,Probe2_ = 0.52) roughly coincide with those extrapolated from the constrained double exponential fitting with *τ_D,_*_XXX_; *τ_D,_*_YYY_ values proper of the correct genotype, but are very different from those yielded by all the other constrained fits fulfilling the *f*_XXX_ and *f*_YYY_ self-consistency criteria (see last columns of Tables 4 and 5 for the DQB1-0201/DQB1-0502 sample).

In Fig. 5, the rectangles surrounding the points indicate the confidence intervals (±σ) in the determination of <*τ_D,Probe2_*> (rectangle base) and <*τ_D,Probe1_*> (rectangle height), respectively. Thus, superposition of two or more rectangles would denounce ambiguous tagging of the corresponding genotypes. As it is apparent from Fig. 5, this situation holds for no genotype. This demonstrates our ability of recognizing any possible genotypic variant of the DQB1 trait encompassing codons 52–57 through combined analysis of the TCSPC data obtained with Probe1 and Probe2 within a confidence of one standard deviation. It must be said that such a degree of confidence is rather poor for a clinical test, corresponding to a probability to measure a <*τ_D_*> value outside the rectangles >0.33. However, although not decisive, the above discussed method might be exploited as a preliminary screening tool, by virtue of its applicability on negligible amounts of untreated cell lysate, which constitutes a unique prerequisite for achieving superior readiness and cost-effectiveness with respect to traditional, PCR-based genotyping techniques. Moreover, if we limit our analysis to selected genotypes of cardinal clinical relevance, discrimination is feasible to a much higher degree of confidence. Namely, the genotypes DQB1-0201/DQB1-0201, DQB1-0201/DQB1-0302, and DQB1-0402/DQB1-0402, which are correlated with the maximum risk factors for celiac disease and IDDM, for Graves disease, and for biliary cirrhosis, respectively, are unambiguously typed even if the required confidence interval is extended to ±3σ, corresponding to a probability to measure a <*τ_D_*> value outside the rectangles <0.003. Furthermore, with the same degree of confidence the ambiguity in recognition of DQB1-0602/DQB1-0602 genotype, encoding enhanced proneness to multiple sclerosis, sarcoidosis, and narcolepsy, involves three other possible genotypes only, namely DQB1-0502/DQB1-0503, DQB1-0602/DQB1-0501, and DQB1-0503/DQB1-0402. Finally, the clustering properties inherited by the one-dimensional <*τ_D,Probe1_*> topology are conserved also by the distance distributions given by the two-dimensional Euclidean metrics applied on the (<*τ_D,Probe1_*>; <*τ_D,Probe2_*>) vectors. As a consequence, the genotypes originally comprehended within the green cluster, and predisposing to Graves and celiac disease, remain recognizable within the confidence interval of ±2σ, with the only ambiguity between the pathogenically relevant genotype DQB1-0201/DQB1-0502 and the non-predisposing genotype DQB1-0302/DQB1-0301. Obviously, removal of such degeneracy by submission of the patients suspected to bear the former genotype to further genetic screening with more refined techniques would be much easier than ab-initio traditional genotyping. The same can be said for the blue cluster, for which extension of the confidence interval to ±2σ only introduces ambiguity in the recognition of the pathogenically relevant genotypes DQB1-0302/DQB1-0402 and DQB1-0302/DQB1-0502, whose rectangles partially superimpose to those of the non-predisposing genotypes DQB1-0501/DQB1-0501 and DQB1-0501/DQB1-0503, respectively.

## Conclusions

We designed two oligonucleotide probes specific for the highly polymorphic trait of the DQB1 gene encompassing codons 52–57, dual-labelled at their opposite ends with a fluorescence resonance energy transfer donor-acceptor pair. A full characterization of the fluorescence response of the donor when the probes are made to hybridize to oligonucleotide mixtures mimicking all the possible genotypes of that trait (8 homozygous and 28 heterozygous) was undertaken. We demonstrated that each genotype can be effectively tagged by the fluorescence decay constants extrapolated from the time-correlated single-photon counting data obtained with both probes. The probability of misassignments is >5% (corresponding to a Δ*τ_D_*/2σ value <2) for 18 out of 36 genotypes. We are aware that such a poor degree of reliability in the attribution of genotypes makes the method unfit to serve as a self-sufficient diagnostic tool in clinical practice. Nonetheless, the readiness and cost-effectiveness of the method, combined with its proneness to be performed on untreated cell lysates, make it a valuable alternative to current genotyping techniques for preliminary wide-range population screening for genetic susceptibility to autoimmune diseases as severe as multiple sclerosis, celiac disease, narcolepsy, diabetes and several others.

## Supporting Information

File S1 **Details on constrained double-exponential fit procedure applied to DQB1-0201/DQB1-0502.**
DOCX
