# Supplementary material for: Full Genotyping of a Highly Polymorphic Human Gene Trait by Time-Resolved Fluorescence Resonance Energy Transfer
Source: PLoS One. 2014 Sep 12;9(9):e107310. doi: 10.1371/journal.pone.0107310 (PMC4162610; doi:10.1371/journal.pone.0107310)
Supplement: File S1 — Details on constrained double-exponential fit procedure applied to DQB1-0201/DQB1-0502. DOCX [file pone.0107310.s001.docx]

Supporting information File S1. Details on constrained double-exponential fit procedure applied to DQB1-0201/DQB1-0502.

In Tables S1 and S2 the *f*_XXX_ and *f*_YYY_ derived for Probe1 and Probe2, respectively, are reported for an exemplary dataset, *i.e.* DQB1-0201/DQB1-0502. For ease of reading, the first allele to be reported, DQB1-0XXX, is always the least mismatching with respect to the probe, i.e. the one forming the more stable duplex. By analyzing the parameters derived from the constrained fits, it is apparent that forcing the fit to converge to most of the possible *τ_D,_*_XXX_; *τ_D,_*_YYY_ pairs results in physically unacceptable results. Namely, for 16 out of 28 genotypes with Probe1 and for 13 genotypes with Probe2 one of the initial amplitudes assumes a negative value (the pertaining genotypes are evidenced by arrows at the right of the genotype labels in Tables S1 and S2). Moreover, for 5 genotypes with Probe1 and 5 with Probe2, *f*_XXX_ < *f*_YYY_ which conflicts with the superior thermodynamic stability of the probe/DQB1-0XXX duplex (the pertaining genotypes are evidenced by arrows at the left of the genotype labels in Tables S1 and S2). Moreover, in both the above instances, if the initial amplitudes are forced to fulfil the physical significance requirements (i) *f*_XXX_ ; *f*_YYY_ > 0 and (ii) *f*_XXX_ > *f*_YYY_, the resulting fits aare notably less accurate than that obtained with the single-exponential model (see the χ^2^ values reported in the last columns of Tables S1 and S2).

Table S1. Initial amplitudes yielded by fitting the data obtained for the sample containing Probe1 and the target-oligonucleotide mixture 0201/0502 to a two-exponential model with decay times constrained to the values measured for the alleles indicated in first column, as reported in Table 2. In the last column the χ^2^ values derived from the fitting by imposing the self-consistency conditions to the amplitude values (i) and (ii) detailed above are reported. The χ^2^ value obtained for unconstrained single-exponential fit is 0.9808.

| **Genotype** | **f_xxx_ × 10^4^** | **f_YYY_ × 10^4^** | **χ^2^ of fit with f_XXX_ and f_YYY_ forced to fulfil conditions (i) and (ii)** |
| --- | --- | --- | --- |
| 0201/0302 ← | -1.25 | 7.25 | 1.0611 |
| → 0201/0301 | 2.25 | 3.77 | 0.9894 |
| → 0201/0503 | 2.92 | 3.09 | 0.9814 |
| → 0201/0402 | 2.52 | 3.50 | 0.9842 |
| → 0201/0501 | 1.66 | 4.35 | 1.0027 |
| **0201/0502** | 3.14 | 2.88 | 0.9791 |
| → 0201/0602 | 2.89 | 3.13 | 0.9816 |
| **0302/0301** | 4.65 | 1.36 | 0.9807 |
| **0302/0503** | 5.07 | 0.94 | 0.9796 |
| **0302/0402** | 4.84 | 1.17 | 0.9796 |
| **0302/0501** | 4.13 | 1.88 | 0.9808 |
| **0302/0502** | 5.18 | 0.83 | 0.9796 |
| **0302/0602** | 5.06 | 0.95 | 0.9806 |
| 0301/0503 ← | 16 | -10 | 1.0733 |
| 0301/0402 ← | 35 | -29 | 1.0733 |
| 0301/0501 ← | -11 | 17 | 1.0420 |
| 0301/0502 ← | 13 | -7.17 | 1.0733 |
| 0301/0602 ← | 17 | -11 | 1.0733 |
| 0503/0402 ← | -19 | 25 | 1.1664 |
| 0503/0501 ← | -4.05 | 10 | 1.0964 |
| 0503/0502 ← | 44 | -38 | 1.2233 |
| 0503/0602 ← | -286 | 292 | 1.2181 |
| 0402/0501 ← | -6.77 | 13 | 1.0592 |
| 0402/0502 ← | 17 | -11 | 1.1176 |
| 0402/0602 ← | 27 | -21 | 1.1176 |
| 0501/0502 ← | 9.21 | -3.20 | 1.0173 |
| 0501/0602 ← | 10 | -4.2 | 1.0173 |
| 0502/0602 ← | -33 | 39 | 1.2594 |

Table S2. Initial amplitudes yielded by fitting the data obtained for the sample containing Probe2 and the target-oligonucleotide mixture 0201/0502 to a two-exponential model with decay times constrained to the values measured for the alleles indicated in first column, as reported in Table 2. In the last column the χ^2^ values derived from the fitting by imposing the self-consistency conditions to the amplitude values (i) and (ii) detailed above are reported. The χ^2^ value obtained for unconstrained single-exponential fit is 1.0143.

| **Genotype** | **f_xxx_ × 10^4^** | **F_YYY_ × 10^4^** | **χ^2^ of fit with f_XXX_ and f_YYY_ forced to fulfil conditions (i) and (ii)** |
| --- | --- | --- | --- |
| → 0503/0602 | 4.26 | 5.62 | 1.0258 |
| **0503/0301** | 5.47 | 4.42 | 0.9954 |
| **0503/0402** | 7.17 | 2.75 | 0.9865 |
| → 0503/0501 | 4.07 | 5.82 | 1.0410 |
| **0503/0502** | 6.73 | 3.18 | 0.9895 |
| 0503/0302 ← | 22 | -12 | 2.7218 |
| 0503/0201 ← | 17 | -6.68 | 2.7218 |
| 0602/0301 ← | 25 | -15 | 2.1411 |
| 0602/0402 ← | 14 | -3.96 | 2.1411 |
| 0602/0501 ← | -115 | 125 | 2.0506 |
| 0602/0502 ← | 15 | -5.40 | 2.1411 |
| **0602/0302** | 6.97 | 2.93 | 1.0094 |
| **0602/0201** | 7.56 | 2.34 | 0.9925 |
| 0301/0402 ← | 19 | -8.75 | 4.1847 |
| 0301/0501 ← | -13 | 22 | 2.8988 |
| 0301/0502 ← | 23 | -14 | 4.1847 |
| **0301/0302** | 5.88 | 4.03 | 1.0057 |
| **0301/0201** | 6.59 | 3.32 | 0.9864 |
| 0402/0501 ← | -3.53 | 13 | 6.9334 |
| 0402/0502 ← | -42 | 52 | 14.0325 |
| → 0402/0302 | 4.07 | 5.88 | 1.2963 |
| → 0402/0201 | 4.83 | 5.13 | 1.0152 |
| 0501/0502 ← | 15 | -4.77 | 1.9641 |
| **0501/0302** | 7.13 | 2.76 | 1.0099 |
| **0501/0201** | 7.70 | 2.19 | 0.9933 |
| → 0502/0302 | 4.57 | 5.36 | 1.0295 |
| **0502/0201** | 5.34 | 4.61 | 0.9789 |
| 0302/0201 ← | 37 | -27 | 6.3085 |
